# Cooperativity in Sorption Isotherms

**DOI:** 10.1021/acs.langmuir.3c01243

**Published:** 2023-09-22

**Authors:** Seishi Shimizu, Nobuyuki Matubayasi

**Affiliations:** † York Structural Biology Laboratory, Department of Chemistry, University of York, Heslington, York, YO10 5DD, U.K.; ‡ Division of Chemical Engineering, Graduate School of Engineering Science, Osaka University, Toyonaka, Osaka 560-8531, Japan

## Abstract

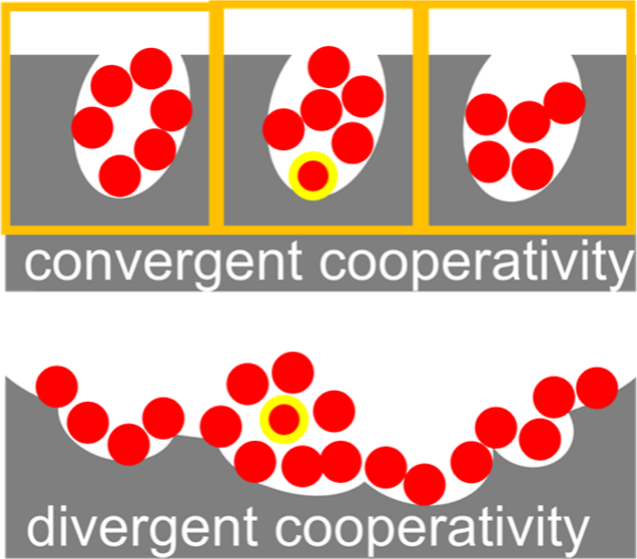

We present a general theory of cooperativity in sorption isotherms that can be applied to sorbent/gas and sorbent/solution isotherms and is valid even when sorbates dissolve into or penetrate the sorbent. Our universal foundation, based on the principles of statistical thermodynamics, is the excess number of sorbates (around a probe sorbate), which can capture the cooperativities of sigmoidal and divergent isotherms alike via the ln–ln gradient of an isotherm (the excess number relationship). The excess number relationship plays a central role in deriving isotherm equations. Its combination with the characteristic relationship (i.e., a succinct summary of the sorption mechanism via the dependence of excess number on interfacial coverage or sorbate activity) yields a differential equation whose solution is an isotherm equation. The cooperative isotherm equations for convergent and divergent cooperativities derived from this novel method can be applied to fit experimental data traditionally fitted via various isotherm models, with a clear statistical thermodynamic interpretation of their parameters..

## Introduction

**Figure 1:**
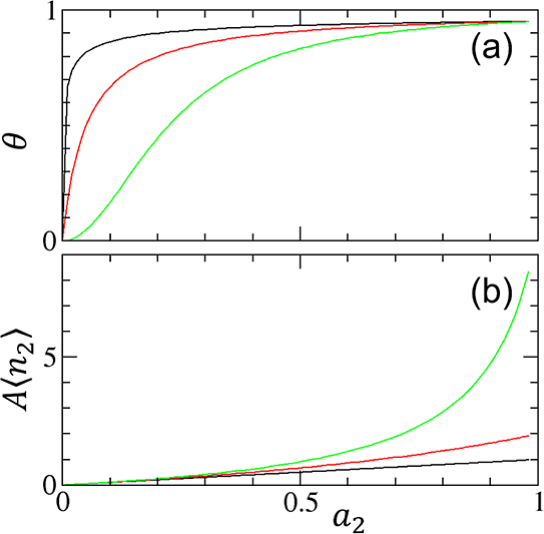
(a) Convergent cooperativity. The fractional coverage θ of the cooperative isotherm (eq 7) is plotted against the activity of sorbate (*a*_2_) with the parameters *A*_*m*_ = 20 and *m* = 0.5 (black), 1.0 (red), and 2.0 (green). (b) Divergent cooperativity. The normalized amount of sorption (*A*⟨*n*_2_⟩) of the AB isotherm (eq 14b) is plotted against *a*_2_ with the parameters *K*_b_ = 0 (black), 0.5 (red), and 0.9 (green).

A steep increase in the sorption isotherm is observed when sorbates, already sorbed at the interface, bring in more sorbates via attractive interactions. This is called cooperative sorption, which may be classified in this paper into the following two categories:

A. Convergent (sigmoidal) cooperativity, such as the IUPAC Types IV and V,^1^ observed for microporous and mesoporous materials (Figure 1a).
B. Divergent cooperativity, such as the IUPAC Types II and III,^1–5^ observed for “non-porous or macroporous surfaces which interact very weakly with adsorbate molecules”^3^ and “[f]oods that are rich in soluble compounds such as sugars”^4^ (Figure 1b).

**Figure 2:**
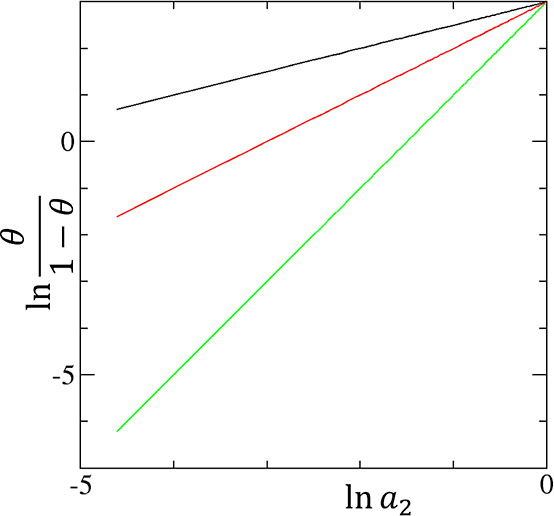
Linearized Hill plot for the cooperative isotherm (eq 13a) for convergent cooperativity (Figure 1a) with the parameters *A*_*m*_ = 20 (i.e., the intercept of the plot) and the gradient of the plot, *m* = 0.5 (black), 1.0 (red), and 2.0 (green).

Convergent cooperativity was first formulated by Hill for oxygen binding on hemoglobin.^6^ From an experimental ligand-binding isotherm, cooperativity underlying binding can be quantified straightforwardly using the linearized Hill plot (Figure 2).^7,8^ However, since the linearized Hill plot presupposes the saturation of binding isotherms at large ligand concentration (Figure 1a), it cannot be applied to the divergent cooperativity to quantify its underlying cooperativity; divergent cooperativity was captured, for example, by the promotion of sorption on the side of the primary sorbate.^9,10^ How can we quantify the sorption cooperativity for divergent isotherms in a manner common to convergent isotherms? Is there any universal measure of sorption cooperativity that encompasses both convergent and divergent cooperativities?

The first aim of this paper is to establish a universal principle of sorption cooperativity that can be applied to both convergent and divergent isotherms. This can be achieved by the fluctuation sorption theory, a model-free theory founded directly on the statistical thermodynamic fluctuation theory.^11–14^ The universal applicability of this theory to any interfacial porosity or geometry contrasts with the previous approaches based on (i) sorption models that assume binding sites, binding constants, and adsorption layers (such as the Langmuir,^15,16^ BET,^17,18^ and GAB^19–21^ models) or (ii) equations of states assumed for the spreading pressure (such as the Volmer,^22^ Hill-de Boer,^21,23^ and Guggenheim^19^ models). Our theory, in contrast, is based on the excess number of sorbates around a probe sorbate, which can be evaluated directly from the gradient of an isotherm (i.e., the excess number relationship).^11–14^ In this paper, the validity of the excess number relationship will be extended to the cases when sorbate and solvent molecules dissolve into or penetrate the sorbent.^24,25^ We will show that the excess number can capture convergent and divergent cooperativities alike.

The second aim of this paper is to derive an equation for cooperative isotherm applicable both for sorbent/gas and sorbent/solution systems based directly on the excess number relationship.^11,12^ This can be achieved by overcoming the two-fold limitations of our recent papers.^24,25^ First, our cooperative isotherm from statistical thermodynamics, despite its capacity to fit Types IV–VI isotherms, has been limited to solid/gas sorption.^24,25^ Cooperative sorption has also been observed in sorbent/solution interfaces and makes an appearance in isotherm classifications (such as Type S by Giles et al.^26–28^ and Type b by the 1986 IUPAC report^29^). Second, the solid/gas cooperative isotherm was derived by postulating the existence of statistically independent patches of microscopic sizes (such as pores and crevices) that constitute the interface.^24,25^ However, the relationship between this postulate and the excess number relationship has remained obscure due to the mathematically involved nature of the derivation.^24,25^ These two-fold limitations will be overcome in this paper through a novel, systematic method to derive isotherm equations via differential equations, based directly on the excess number relationship. We will demonstrate that this method, an alternative to the common approaches (e.g., based on site-specific models^15–21^ or equations of states for the spreading pressure^11–14^), is versatile and is capable of deriving widely varying types of isotherms.

The third aim of this paper is to clarify the physical meaning of negative cooperativity in sorption. In sorption, negative cooperativity is often described using the Freundlich isotherm model^30,31^ which, although initially proposed as an empirical model, corresponds at dilute sorbate concentrations to a third case of the Hill model^6^ (positive, zero, and negative cooperativities) where the reduction in subsequent ligand affinities is caused by the first ligand (see Figure 1a).^7,30,31^ However, our recent statistical thermodynamic cooperative isotherms, while successful in modeling isotherms with positive and zero cooperativities, are incapable of capturing negative cooperativity.^24,25^ Our third aim, therefore, is to clarify the origin of negative sorption cooperativity for both sorbent/gas and sorbent/solution sorption based directly on the excess number relationship (the first aim) and the novel method for deriving sorption isotherms (the second aim).

## Theory

### Sorbent/Gas Sorption

#### Setup

The fluctuation sorption theory is founded on a statistical thermodynamic generalization of the Gibbs isotherm, applicable to any interfacial geometry and porosity,^11^ based on a postulate on the finite-ranged nature of an interface.^11–13,32^ Following the statistical thermodynamic notation,^11–13,32^ we denote sorbate molecule as species 2, its number at the interface (within the volume *v*) as *n*_2_^*^, and its activity as *a*_2_. The sorption isotherm is the dependence of sorbate surface excess, ⟨*n*_2_^*^⟩–⟨*n*_2_^I^⟩–⟨*n*_2_^II^⟩, on *a*_2_, where ⟨⟩ represents ensemble averaging, and *n*_2_^I^ and *n*_2_^II^ are the number of sorbates in the sorbent and vapor reference states (with the same volume *v*) as the interface.^11–13,24,32,33^ Since the sorbate is dilute in the reference states I and II, the surface excess can be approximated by “the amount of sorption”, ⟨*n*_2_^*^⟩, via ⟨*n*_2_^*^⟩–⟨*n*_2_^I^⟩–⟨*n*_2_^II^⟩ ≃ ⟨*n*_2_^*^⟩.^11–13,24,32,33^ Following our recent papers, we shall omit * for sorbent/gas isotherms from now onward.^11–13,24,32,33^

#### Excess Number Relationship

Here, we summarize the fundamental equation of the fluctuation sorption theory. Sorbate–sorbate interaction, which takes place at the interface, can be evaluated from an isotherm, using the excess number of sorbates around a probe sorbate, *N*_22_, defined in terms of the sorbate number correlation
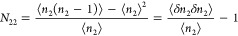
1where δ*n*_2_ = *n*_2_ – ⟨*n*_2_⟩ as the deviation of *n*_2_ from the mean. A favorable sorbate–sorbate interaction drives up *N*_22_, while repulsion, including the excluded volume effect, drives it down. The excess number can be calculated straightway from the gradient of an isotherm via^11–13^
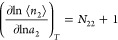
2This equation is the fundamental relationship for sorbent/gas sorption, which can reveal the underlying sorbate–sorbate interaction from an experimental isotherm.

#### Excess Sorbate Cluster Number

Our fundamental equation (eq 2), termed the excess number relationship, can be interpreted in an alternative, equivalent manner. Counting the probe sorbate itself (i.e., the term 1 on the right-hand side), *N*_22_ + 1 can also be interpreted as the *excess* sorbate cluster number.^11–13^ Here, we emphasize the importance of the term “excess” in the interpretation of *N*_22_ + 1 [an increase in the amount of sorption (hence in the fractional saturation of an interface) may not necessarily lead to a larger sorbate cluster size; a greater fractional saturation may lead to diminishing sorbate–sorbate correlation, as will be shown in Results and Discussion]. We will demonstrate that the relationship between the interfacial filling and excess sorbate number will play a key role in deriving the isotherm equations.

#### Cooperative Isotherm

As emphasized in the Introductionsection, rederiving an isotherm may lead to new insights into the sorption mechanism. Here, we present a facile rederivation of the cooperative isotherm, which has a clear and direct link to the foundation of the fluctuation sorption theory (eq 2). Our first step is to simplify eq 2 using the inhomogeneous solution theory,^34,35^ as
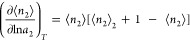
3awhere
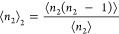
3bThe left-hand side of eq 3b represents the ensemble average of sorbate number in an inhomogeneous ensemble, namely, the conditional ensemble average in the presence of the probe sorbate 2 fixed at the origin.^36,37^ Our second step for deriving the cooperative isotherm is to establish how ⟨*n*_2_⟩_2_ + 1 – ⟨*n*_2_⟩ in eq 3a changes with ⟨*n*_2_⟩. This can be achieved by postulating that the interface is composed of *N* statistically independent microscopic patches, such as pores or crevices.^24,25^ This postulate was implemented also in our previous papers,^24,25^ yet without a clear link to the excess number relationship (eq 2), i.e., the foundation of the fluctuation sorption theory. Our intuitive implementation of this postulate in the following section will help clarify this link. Let the number of sorbates within a microscopic patch be denoted by *v*_2_ hereafter and the probe sorbate be in one of the patches. With this setup, the statistical independence is equivalent to the sorbate–sorbate correlation being restricted within the same patch. Consequently, the mean sorbate number within the patch is conditional to the presence of the probe and is denoted as ⟨ν_2_⟩_2_. It deviates from that of other *N* – 1 patches, ⟨ν_2_⟩, that do not feel the effect of the probe. Hence, ⟨*n*_2_⟩_2_ of the total interface can be expressed as
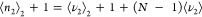
4aWe can simplify eq 4a by introducing
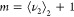
4bas the excess sorbate cluster number in the patch that contains the probe. In the following development of our theory, *m* is assumed to be a constant, which does not depend on *a*_2_ or ⟨*n*_2_⟩; it is the extrapolation of ⟨ν_2_⟩_2_ to *a*_2_ = 0, rather than the true limiting value at *a*_2_ → 0, as shown in Supporting Information: **Limiting excess number and cooperativity**. Having evaluated ⟨*n*_2_⟩_2_ + 1 within ⟨*n*_2_⟩_2_ + 1 – ⟨*n*_2_⟩ in eq 3a via eqs 4a and 4b, now we turn to ⟨*n*_2_⟩, rewriting it also using the postulate of statistically independent patches. By definition, none of the patches that constitute ⟨*n*_2_⟩ contains any probe sorbate. This means that all of the patches are statistically equivalent. Consequently
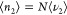
4cCombining eqs 4a–4c, we obtain
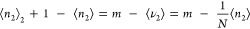
5where, in the final step, eq 4c is used to eliminate ⟨ν_2_⟩. Equation 5 is the characteristic relationship for this sorption isotherm: the excess sorbate cluster number [*N*_22_ + 1 = ⟨*n*_2_⟩_2_ + 1 – ⟨*n*_2_⟩ via eqs 2 and 3b] decreases linearly with the amount of sorption, ⟨*n*_2_⟩. Combining eqs 3a and 5, a nonlinear differential equation can be derived
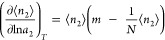
6aSolving eq 6a is facilitated by introducing the fractional saturation
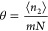
6bthrough which eq 6a can be rewritten as
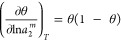
6cIntegrating eq 6c via the separation of variables and partial fraction decomposition yields
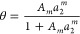
7where *A*_*m*_ is an integration constant. In our previous paper, we have shown that −*RT* ln *A*_*m*_ signifies as the free energy of transferring *m* sorbate molecules cooperatively from saturated vapor to the interface^24^ (note that eq 7 is valid for finite *a*_2_ yet modification is required to be exact at *a*_2_ → 0, as shown in the Supporting Information: **Limiting excess number and cooperativity**). To summarize, we have discovered a facile and insightful rederivation of the statistical thermodynamic cooperative isotherm, whose simplicity and insight should be contrasted with significant mathematical work in our previous paper.^24^ The new physical insight (eq 5), which is a concise expression of the statistical independence of small patches, led to this simple derivation.

### Sorbent/Solution Sorption

#### Setup

Here, we generalize our discussion of sorbent/gas isotherms to sorbent/solution isotherms. We follow the standard treatment of sorbent/solution sorption isotherms, which neglects ⟨*n*_2_^I^⟩, i.e., the number of sorbates inside the sorbent (reference system I), while incorporating ⟨*n*_2_^II^⟩ (i.e., the number of sorbates in the solution, denoted as the reference system II^29^). Unlike sorbent/gas systems, the solvent (species 1) must also be taken into account. There are two common quantities for sorption that are interrelated.^29^ The first is Γ_2_^(1)^, the “relative surface excess of 2 with respect to 1″,^29^ which is related to the amounts of solvent ⟨*n*_1_^*^⟩ and sorbate ⟨*n*_2_^*^⟩ at the interface, via
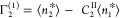
8awhere *C*_2_^II^ = ⟨*n*_2_^II^⟩/⟨*n*_1_^II^⟩ is the sorbate/solvent mole ratio in the solution phase. The second is the *reduced* surface excess, Γ_2_^(*n*)^, which can be obtained directly from experiments^29^ and is related to Γ_2_^(1)^ via^29^
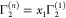
8bwhere *x_1_* is the mole fraction of the solvent species in the solution phase (II). These quantities are applicable to molecular and weak electrolyte sorbates.^29^ A sorbent/solution isotherm, in principle, is the dependence of Γ_2_^(1)^ or Γ_2_^(*n*)^ on the sorbate activity, *a*_2_. However, in practice, the sorbate concentration in the solution phase (such as *x*_2_^II^, the mole fraction of sorbate) is used commonly as the variable instead of *a*_2_.^26,27,29,38,39^

#### Fluctuation Theory for Sorbent/Solution Isotherms

Here, we generalize our excess number relationship for the sorbent/gas theory on excess sorbate number (eq 2) to sorbent/solution isotherms. To carry this out directly would incur significantly cumbersome algebra. However, as has been detailed in Supporting Information: **Derivation of the cooperative isotherm for the sorbent/solution interface**, the ensemble invariance of mole ratio fluctuations^40,41^ provides a practical route to simplification. The technicality of the derivation is detailed in the Supporting Information; its key idea is statistically transforming the interfacial and the solution reference ensembles, originally defined under constant μ_1_, to constant *n*_1_ ensembles for the ease of calculation.^40,41^ This leads to the following generalization of the sorbent/gas theory (eq 2) to the sorbent/solution interface
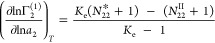
9awhere *N*_22_^*^ and *N*_22_^II^ are the sorbate excess numbers, defined in the constant *n*_1_ ensemble at the interface and in the solution reference system, respectively. To simplify the mathematical expression, we have introduced the exchange constant, *K*_e_, defined as
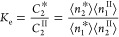
9b*K*_e_ has a simple physical interpretation, which corresponds to an exchange of “a sorbate in the solution + a solvent at the interface” with “a sorbate at the interface + a solvent in the solution”. The presence of *K*_e_ in eq 9a comes from the fact that this exchange equilibrium *K*_e_ is part of the sorbent-solution sorption process. Equation 9a is a significant new result valid for any sorbent/sorption isotherms as long as sorbate does not penetrate the sorbent. Note that our sorbent-solution theory (eq 9a) is a generalization of our sorbent-gas theory (eq 2). This can be demonstrated by reducing eq 9a to the form mathematically identical to that of sorbent/gas (eq 2) under *K*_e_ ≫ 1, which is equivalent to *C*_2_^*^ ≫ *C*_2_^II^ [where *C*_2_^*^ = ⟨*n*_2_^*^⟩/⟨*n*_1_^*^⟩ is the mole ratio at the interface signifying a very strong sorption]. Under this condition, Γ_2_^(1)^ = ⟨*n*_1_^*^⟩(*C*_2_^*^ – *C*_2_^II^) ≃ ⟨*n*_2_^*^⟩, hence
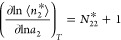
9cwhich is mathematically analogous to eq 2.

#### Cooperative Sorbent/Solution Isotherm

We have shown that the excess number relationships for sorbent/gas isotherms (eq 2) and sorbent/solution isotherms under strong sorption (eq 9c) obey the mathematically analogous fundamental equation. For this reason, we shall drop the superscript * in eq 9c from now onward, unless there is a need to specify a sorbent/solution interface. Consequently, we can follow the same argument (eqs 3a–6c) to derive the cooperative sorbent/solution isotherm for strong sorption, i.e., eq 7.

#### Connection to χ

*N*_22_ for the sorbent/solution interface in eq 9a (i.e., * and II) has been defined in the constant *n*_1_ ensemble for mathematical simplicity.^40,41^ Note that this ensemble is different from the constant μ_1_ ensemble, in which the relative and reduced surface excesses have been defined (eqs 8a and 8b). However, conversion from the constant *n*_1_ to constant μ_1_ ensemble is straightforward via the statistical variable transformation (Supporting Information: **Derivation of the cooperative isotherm for the sorbent/solution interface**), which yields
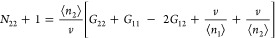
10Equation 10 has a clear physical interpretation. *N*_22_ + 1, when viewed in the constant μ_1_ ensemble, represents the net self-interaction (i.e., the difference between self-interactions, *G*_11_ and *G*_22_, and mutual interaction, *G*_12_). The common measure for net self-interaction is the Flory χ parameter, which is restricted to the lattice model of solutions,^42–44^ yet can be generalized beyond the lattice model as
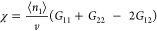
11based on a correspondence in activity coefficients between the lattice model and the Kirkwood–Buff theory of solutions.^45^ Using eq 11, *N*_22_ can be expressed as
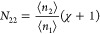
12This marks a departure from the sorbent/gas interface, in which only the sorbate–sorbate interaction contributes to *N*_22_ + 1. With this interpretation in mind, eq 9a is a general relationship applicable to all sorbent/solution interfaces, enabling a model-free elucidation of the sorption mechanism underlying an isotherm for which the ln–ln plot of the isotherm still plays a key role.

#### Penetration of Sorbate and Solvent into the Sorbent

We have derived our fundamental relationships (eq 2 for sorbent/vapor and eqs 9a for sorbent/solution) in the framework of sorption founded on the generalized Gibbs isotherm.^11^ However, we have shown in the Supporting Information: **Penetration of solvent and sorbate into sorbent**, based on a pair of the Gibbs–Duhem equations for the system and the reference state under constant temperature and pressure,^46^ that our fundamental relationships are valid even when the solvent and sorbate dissolve into or penetrate the sorbent. Thus, the same isotherm equations can be applied to adsorption isotherms and “solubility isotherms”^47^ alike.

## Results and Discussion

### Cooperativity of Sorption Isotherms

#### Convergent Cooperativity as Diminishing Sorbate Excess Number

**Figure 3:**
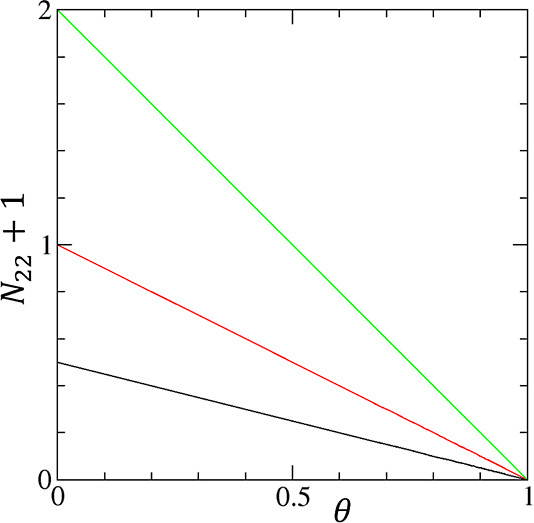
Excess sorbate cluster number, *N*_22_ + 1, against the fractional saturation θ for the cooperative isotherm (eq 13b) for convergent cooperativity (Figure 1a) with the parameters *m* = 0.5 (black), 1.0 (red), and 2.0 (green).

**Figure 4:**
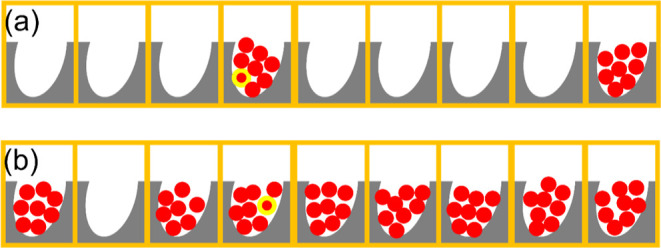
Schematic diagram for the mechanism of convergent cooperativity (Figure 1a), rationalizing how the statistically independent patches of microscopic sizes (orange boxes) lead to the decrease of sorbate excess cluster number *N*_22_ + 1 as the increase of fractional coverage [from (a) to (b)]. (a) At low fractional coverage, filled patches are distributed sparsely. Therefore, the sorbate cluster around the probe is *localized*, leading to a large *excess* cluster numbers. (b) At high fractional coverage, filled patches are more common than empty patches, hence the presence of the probe sorbate does not make the patch more populated, hence the *excess* cluster number is closer to zero.

In the Introduction section, we have classified sorption cooperativity into convergent and divergent subcategories (Figure 1). In the Theory section, we have derived the cooperative isotherm equation applicable to convergent cooperativity by a novel approach: a differential equation from fluctuation theory in combination with the statistical independence of the microscopic patches that constitute an interface. The resultant cooperative isotherm (eq 7) can also be expressed in the following linearized form
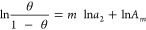
13aEquation 13a is mathematically identical to the linearized Hill plot (Figure 2). However, its foundation is different from that of the Hill model. Unlike the Hill model,^7^ the fluctuation theory (eq 3a) does not involve any assumptions on the binding sites, stoichiometry, or chemical ligand linkage.^7,48,49^ Our sole postulate, instead, is the statistical independence of interfacial patches with constant *m*, which was shown to cause a linear reduction of the sorbate cluster number with θ (Figure 3)
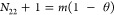
13bwhich applies both to sorbent/gas and sorbent/solution isotherms (hence the superscript *, used for sorbent/solution systems to denote the interface, was dropped). Here, why excess sorbate cluster number *N*_22_ + 1 decreases linearly with θ can be understood from the definition of *N*_22_ as the *excess* number (Figure 4). At low fractional saturation, the presence of the probe molecule affects the distribution of other sorbates (Figure 4a). However, at high fractional saturation, the interface is packed already with sorbate regardless of the presence of the probe sorbate; hence, the number correlation goes down (Figure 4b). Such a behavior of the characteristic relationship reflects the geometric organization of the surface. Thus, *N*_22_ + 1 diminishing with θ is the cause of the convergent cooperativity, which captures the effect of surface geometry on sorbate interactions.

#### Divergent Cooperativity as the Growing Sorbate Cluster

**Figure 5:**
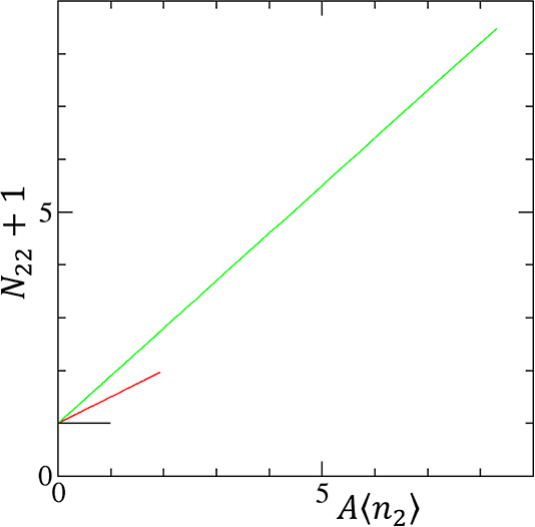
Excess sorbate cluster number, *N*_22_ + 1 (eq 14c), against the normalized amount of sorption *A*⟨*n*_2_⟩ for the AB isotherm (eq 14b) for divergent cooperativity (Figure 1b) with the parameters *K*_b_ = 0 (black), 0.5 (red), and 0.9 (green).

**Figure 6:**
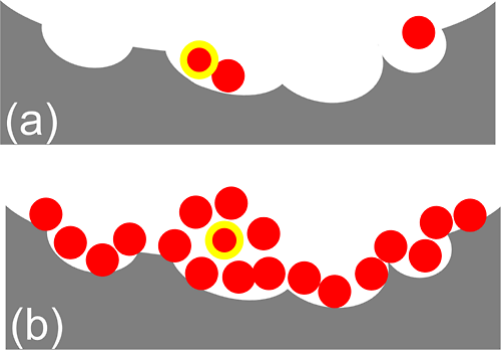
Schematic diagram for the mechanism of divergent cooperativity (Figure 1b), rationalizing how the increase in the amount of sorption from (a) to (b) leads to the presence of more sorbate molecules around a probe sorbate (red with yellow).

We have shown that the linearized Hill plot for sorption (eq 13a) comes from the statistically independent microscopic patches that lead to diminishing excess sorbate clusters with fractional coverage. Here, we show that the linearized Hill plot (eq 13a) cannot be applied to divergent cooperativity because of its opposite signature, i.e., increasing *N*_22_ + 1 with the amount of sorption. This can be demonstrated most straightforwardly using the AB isotherm (i.e., the simplest type III parameter range of *B* > 0 and *C* = 0 adopted for the ABC isotherm), as^14^
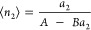
14awhich can be expressed in a normalized manner, as
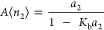
14bwhere *K*_b_ = *B*/*A* can be interpreted as the sorbate–sorbate binding constant in the indefinite binding model.^14^ The excess sorbate cluster number can be calculated by combining eq 14b with eq 2, as
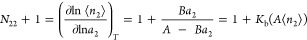
14cThis means that the excess sorbate cluster number increases linearly with the (normalized) amount of sorption, *A*⟨*n*_2_⟩ (Figure 5). The linearly increasing sorbate cluster number contrasts with the convergent cooperativity in Figures 3 and 4. In divergent cooperativity, the interface is not divided into statistically independent microscopic patches. Since the sorbate–sorbate interaction is weak for lower amounts of sorption, the excess sorbate cluster number is small (Figure 6a). However, since the dominant driving force for sorption is the sorbate–sorbate interaction,^14^ a probe sorbate, when the amount of sorption is high, can bring in even more sorbate molecules cooperatively (Figure 6b). Thus, the convergent and divergent cooperativities exhibit an opposite behavior regarding how the sorbate cluster number evolves with sorption.

#### Zero Cooperativity

The cooperative isotherm (eq 7), under *m* = 1, reduces to a form identical to the Langmuir model, which can be understood also from the postulate of statistically independent microscopic patches (see the Theory section). While all of the patches are statistically independent in the absence of a probe (see eq 4c), no additional sorbate can come into the patch that already contains the probe sorbate (eqs 4a and 4b with ⟨ν_2_⟩_2_ = 0). Thus, as we have clarified in our recent papers,^13,14^ sorbate–sorbate exclusion at the interface plays a key role in the Langmuir model.

#### Sorbate Excess Number as the Universal Measure of Cooperativity

We have shown that the difference between convergent and divergent cooperativities comes down to how *N*_22_ + 1 changes with sorption. This means that we should simply adopt *N*_22_ + 1 as the universal measure of sorption cooperativity. Indeed, the excess number relationship between *N*_22_ + 1 and the ln–ln plot of an isotherm (eq 2 for sorbent/gas and eq 9a for sorbent/solution) is universal for any interface. Based on this generalization, we can summarize three different types of cooperativities as.

- positive cooperativity = sorbate–sorbate attraction, *N*_22_ > 0;
- negative cooperativity = sorbate–sorbate repulsion, *N*_22_ < 0;
- zero cooperativity = net zero sorbate–sorbate interaction, *N*_22_ = 0.

### Sorption Isotherms from Differential Equations

The ease and insights with which the cooperative isotherms have been generalized to the sorbent/solution interface owe to the novel approach to deriving sorption isotherms presented in the Theory section, founded directly on the fluctuation sorption theory. The key to our new approach can be summarized as

(1) the excess number relationship as the fundamental equation of the fluctuation solution theory, relating the isotherm gradient [e.g.,  for sorbent/gas (eq 2) and  for sorbent/solution (eq 9a)] to sorbate fluctuation [e.g., *N*_22_ + 1 for sorbent/gas (eq 2) and [*K*_e_ (*N*_22_^*^ + 1) – (*N*_22_^II^ + 1)]/(*K*_e_ – 1) for sorbent/solution (eq 9a)];
(2) a characteristic relationship on sorbate fluctuation, such as its linear decrease with fractional coverage (eq 13b) for convergent cooperativity;
(3) solving a differential equation from (1) and (2) to derive an isotherm.

Our new approach is not restricted to the derivation of cooperative isotherms alone. Other useful isotherms can also be derived by the same approach (Supporting Information: **Isotherms via differential equations**); not only can the already-known statistical thermodynamic isotherms, such as the ABC isotherm (i.e., the model-free generalization of the Langmuir, BET, and GAB models) be derived but also other isotherms that have been proposed previously.^50,51^ Moreover, the AB isotherm for divergent cooperativity (eq 14a) can also be derived, starting from the postulate of linearly increasing sorbate cluster number (Supporting Information: **Divergent cooperativity via the differential equation approach**).

#### Sorbent/Solution Cooperative Isotherm in the Mole Fraction Scale

Sorbate activity, which has a direct relationship with the chemical potential, is a fundamental quantity in the thermodynamics of sorption. However, in practice, *a*_2_ is rarely used in reporting sorbent/solution isotherms. Instead, the sorbate concentration in the solution phase has been used commonly in the experimental literature. Therefore, it is necessary to formulate our theory using the sorbate concentration. Since the cooperative isotherms in sorbent/solution systems fall under the “partially miscible” category (with regard to sorbate and solvent) of isotherm classification with typically very low *x*_2_,^26,27,29^ we can apply Raoult’s Law,^52,53^*a*_2_ ≃ *x*_2_, which transforms the cooperative isotherm (eq 7) into the mole fraction-based form, as
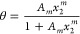
15A generalization to *m* < 1 will be carried out in the next subsection.

#### Statistical Thermodynamic Foundation for Isotherm Classifications

**Figure 7:**
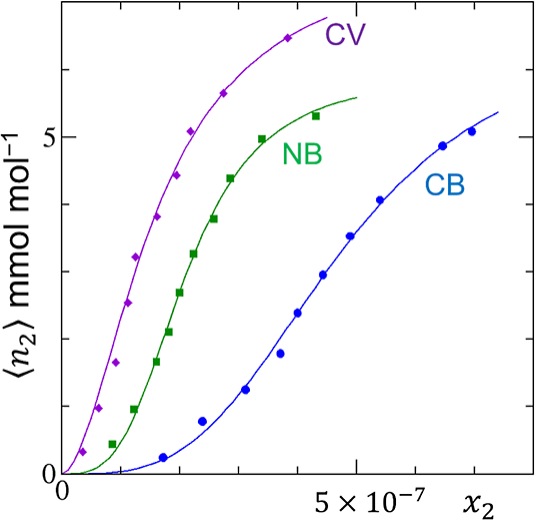
Fitting of the cooperative isotherm (eq 16a) to the experimental adsorption data of cresyl blue (CB), nile blue (NB), and cresyl violet (CV) on cross-linked hydroxamate polymers containing ethylene glycol dimethacrylate (CHP-E) at 25 °C using the data reported by Saraydın et al.^55^ The fitting parameters are summarized in Table 1.

We have recently demonstrated that the two statistical thermodynamic isotherms (the ABC and cooperative) can, in combination, fit all six IUPAC sorbent/gas isotherm types.^14,24,25^ Following this success, here, we show that our generalized isotherms can be applied for sorbent/solution isotherm classifications. Sorbent/solution isotherms are classified into the “fully miscible” and “partially miscible” categories based on the sorbate–solvent miscibility in the solution phase.^29^ Our focus in this paper is the partially miscible category which contains the four main classes of isotherms according to Giles et al.:^27,28,54^ S, L (“Langmuir”), H (“high affinity”), and C (“constant partition”). They are distinguished from one another by the second-order derivative.^28^ Of these classes, the IUPAC report (1986) has identified classes S and L with saturation as “the two extreme forms”.^29^ The classes L, H, and C can be captured by the ABC isotherm when generalized for sorbent/solution. The class S can be modeled successfully by our sorbent/solution cooperative isotherm, as demonstrated using the sorption of dyes from aqueous solutions on a cross-linked polyhydroxamate^55^ (Figure 7 and Table 1). To facilitate fitting and sense checking of the resultant parameters, we have rewritten eq 15 in the following form
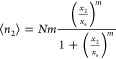
16awhere *A*_*m*_ = *x*_s_^–*m*^ and θ = ⟨*n*_2_⟩/*Nm*. The advantage of this rewriting is two-fold: (1) to facilitate fitting while θ = 1 is hard to locate from the data and (2) to make clear that *x*_s_ corresponds to the point at which the isotherm gradient is the steepest.^25^ Indeed, it is possible to examine the location of *x*_s_ that approximately corresponds to such a point (Table 1). Note that the use of the mole fraction (*x*_2_) for the abscissa may strike as different from the convention (i.e., mole or mass of sorbate per volume). This was necessitated by Raoult’s law for dilute sorbate solutions. As a result, *x*_s_ has a clear physical interpretation. Since −*RT* ln *A*_*m*_ is the free energy of transferring *m* sorbate molecules cooperatively from saturated vapor to the interface,^24^*x*_s_, through its relationship to *A*_*m*_, can be interpreted as
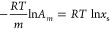
16bwhich shows that *RT* In *x*_s_ is the transfer free energy per sorbate. Thus, together with our previous papers,^24,25^ we have demonstrated that the isotherm fitting can yield parameters with a clear statistical thermodynamic interpretation.

**Table 1:** Fitting Parameters for eq 16a for the Adsorption of Dyes on CHP-E (Figure 7)

sorbate	*N* mmol mol^–1^	*x*_S_	*m*
cresyl blue	6.55	4.72 × 10^–7^	3.36
nile blue	5.91	2.11 × 10^–7^	3.28
cresyl violet	7.84	1.61 × 10^–7^	1.81

### Negative Sorption Cooperativity

#### Differential Equation for Negative Cooperativity

Our argument on the statistically independent patches in the Theory section presupposed a positive cluster number (*N*_22_ + 1 > 0) due to eq 1, even when the probe itself was included in the counting. Therefore, it is necessary to extend our cooperative sorption theory to incorporate negative cooperativity. In the previous subsections, we have established that (i) *N*_22_ < 0 is the measure of negative cooperativity and (ii) isotherms are derived from the excess number relationship (eq 2 for sorbent/vapor and eq 9c for sorbent/solution) that links *N*_22_ to the ln–ln gradient of an isotherm by solving differential equations. Therefore, following the above (i) and (ii), we will construct an isotherm with negative cooperativity. We shall start from the following simplest characteristic relationship for the excess number
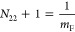
17awith the parameter range of *m*_F_ > 1, which corresponds to *N*_22_ < 0. Just like the parameter *m* in eq 4b, the Freundlich constant *m*_F_ is a constant that does not depend on *a*_2_, *x*_2_, or ⟨*n*_2_⟩. Combining eq 17a with the fundamental excess number relationship (which encompasses eq 2 for sorbent/vapor and eq 9c for a strong surface–sorbate interaction) yields
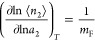
17bwhere *, signifying the interface for sorbent/solution, was omitted. Integrating eq 17b yields
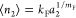
17cwhere *k*_F_ is the integration constant. Equation 17c can be rewritten in the familiar form of the Freundlich model by changing *a*_2_ to *p* (the gas pressure) via *a*_2_ = *p*_2_/*p*_2_^o^ for sorbent/vapor isotherms. For the sorbent/solution isotherms, changing *a*_2_ to the sorbate concentration in the solution phase, *c*_2_, using the dilute ideal condition, *a*_2_ ≃ *x*_2_ ≃ *c*_2_/*c*_1_, yields the common form of the Freundlich model for sorbent/solution interface, as
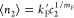
17d

When the sorbate–surface interaction is not as strong, we must base our discussion on eq 9a for the sorbent/solution interface. Our characteristic equation
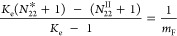
18ain combination with eq 9a yields
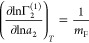
18bwhose integration leads to
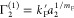
18cThis, under the dilute ideal condition,^52,53^ yields
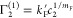
18dwhich is known as the Freundlich model. The Freundlich model is often seen as an indication of interfacial heterogeneity.^56,57^ Such an interpretation, however, is based on an assumption that the interface be comprised of the Langmuir adsorption sites with the distribution in adsorption energies, which, in the case of the Freundlich model, is broad and exponential.^58–62^ In contrast, our theory derives negative cooperativity directly from the excess number relationship, with a clear interpretation, as discussed in the next paragraph.

#### Interactions Underlying the Freundlich Model

In the previous paragraph, we have derived what is known as the Freundlich model simply from the constant, negative sorbate–sorbate excess number, i.e.
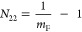
19

Because *m*_F_ > 1, *N*_22_ < 0, which means sorbates are excluded from one another at the interface, reflecting a strong repulsive interaction between sorbates (Table 2 presents a calculation of *N*_22_ from the reported Freundlich constants). Explicit consideration of the surface excess is required for weaker interactions using eq 18a, which shows that *m*_F_ > 1 signifies a weaker self-association at the interface compared to the bulk because *K*_e_ is positive in eq 9b (see eqs 11 and 12). This means that the presence of the interface makes sorbates more separated than that in the bulk. Note, however, that *N*_22_ in the bulk tends to 0 for dilute sorbate concentrations, which again leads to the conclusion that *N*_22_ at the interface is negative, even for weaker sorbate–surface interactions. Thus, sorbate–sorbate repulsion at the interface is the interaction underlying the Freundlich model. We emphasize here that sorbate–sorbate interactions are conditional to the presence of the interface (for example, a strong, site-specific interaction separates the sorbate molecules, acting as sorbate–sorbate repulsion). To summarize, a successful fit by the Freundlich model shows constant negative cooperativity, which reflects the effective sorbate–sorbate repulsion at the interface.

**Table 2:** Sorbate–Sorbate Excess Numbers, *N*_22_, Calculated from the Freundlich Constant from Water on Activated Carbon at 20 °C from the Parameters in ref (74), *m*_F_, via eq 19

dye	*m*_F_	*N*_22_
basic blue 69	1.47	–0.318
basic yellow 21	2.71	–0.631
basic red 22	7.52	–0.867
disperse blue 7	5.00	–0.80
victoria blue	3.11	–0.678
deorlene yellow	6.90	–0.855
telon blue	9.35	–0.893

#### Cooperative Isotherm

Is the cooperative isotherm (eq 15) valid for negative cooperativity? Indeed, the Sips model^63^ is mathematically identical to this case, i.e., eq 15 with *m* < 1. Such an isotherm can be derived by solving the differential equation identical in form to eq 6a, with the only difference of *m* < 1 instead of *m* ≥ 1. This means that the characteristic equation (eq 13b) starts at θ = 0 from sorbate–sorbate exclusion (*N*_22_ = *m* – 1 < 0) which decreases further with the fractional coverage, θ, of the interface. Unlike the *m* ≥ 1 case, the *m* < 1 characteristic relationship (eq 13b) cannot be interpreted using the statistically independent microscopic patches because the presence of a probe sorbate makes *m* < 1 impossible to fulfill. Based on this argument, we conclude that the cooperative isotherm for *m* < 1, known as the Sips model,^63^ signifies the sorbate–sorbate exclusion getting stronger with the fractional coverage, yet cannot be captured by the statistically independent microscopic patches that were useful for *m* ≥ 1.

### Significance

Our novel approach is complementary to the current experimental and computational approaches, providing a link and a common ground between an isotherm (which is macroscopic by nature) and the underlying microscopic interactions (such as van der Waals interactions and Hamaker constants).

#### Experimental Isotherm → Underlying Interactions

The excess number relationship (eqs 9a and 9c) plays a central role in evaluating the sorbate excess numbers, the signature of microscopic interactions underlying an isotherm, from the ln–ln gradient of an experimental isotherm (eqs 9a and 9c). The excess number relationship is also the foundation of deriving isotherm equations, whose parameters signify how the excess number changes with activity or interfacial coverage.

#### Redeployment of Existing Isotherm Models

Evaluating the sorbate excess numbers is crucial for quantifying the interactions underlying an isotherm. Our rederivation of the commonly used isotherms (such as Freundlich and Sips) has opened up a new possibility: the wealth of Freundlich constants available in the literature can be converted straightforwardly (via eq 19) to the sorbate excess numbers, as has already been demonstrated in Table 2. Not only have the empirical models been given a clear interpretation but also be redeployed to yield the underlying sorbate excess numbers as the mechanistic signature of an isotherm.

#### Intermediary between Computational and Experimental Approaches

The major hindrance to a mechanistic understanding of sorption, in our view, has been the disconnect between the isotherm models and computational approaches. Computational approaches, such as the density functional theory^64–66^ and molecular dynamics,^67^ are capable of simulating, based on intermolecular interactions, not only isotherms but also the underlying molecular distribution functions as a route to mechanistic insights.^68–71^ In contrast, the common isotherm models for fitting experimental data are not founded on molecular distribution functions.^14^ Such a long-standing disconnect between experimental and computational approaches has been rectified by the sorbate excess numbers, defined as the integration of the sorbate–sorbate distribution function.^11–13^ Our intention was to consecrate the excess number as the common language shared by theory and the experiment.

#### Temperature Dependence

This paper has focused on excess sorbate numbers based on sorbate number correlations. To understand how sorption changes with temperature, not only the number–number correlation but also the number–energy correlation must be considered. Such an approach by our recent paper^32^ has provided a rigorous theoretical foundation for the adsorption potential theory^72,73^ and shed light on how temperature dependence may be affected by the pore size distribution. However, our previous paper is limited to gas and vapor sorption, and its extension to solid/solution systems is necessary.^32^

## Conclusions

Cooperativity in sorption isotherms (Figure 1) is driven by a strong sorbate–sorbate interaction. This paper has established

i. the universal measure of sorption cooperativity, i.e., the sorbate excess number around a probe sorbate, *N*_22_, which is applicable to sorbent/gas and sorbent/solution isotherms alike;
ii. a general method for deriving sorption isotherms via solving differential equations, set by a combination of the excess number relationship (eqs 2 and 9a) in conjunction with a characteristic relationship (eq 5 and Supporting Information: **Isotherms via differential equations**) describing how *N*_22_ changes with interfacial coverage or sorbate activity;
iii. that our fundamental excess number relationship and isotherms are applicable even when solvent and sorbate molecules dissolve into or penetrate the sorbent (e.g., polymer).

Sorbate excess number can quantify sorption cooperativity for both convergent and divergent isotherms (Figure 1), revealing the sorbate–sorbate interaction that underlies cooperativity [see (i), above]. The characteristic equation for the convergent cooperative isotherm (eq 7, Figure 1a) is the linearly diminishing *N*_22_ with the interfacial coverage (eq 13b) while that for the divergent isotherm (the AB isotherm, eq 14a and Figure 1b) is the linearly increasing *N*_22_ with the sorbate activity (eq 14c), both of which can be derived by solving the differential equation (eqs 2 and 9c). The Freundlich model (eq 17d) can also be derived from the characteristic equation (eq 17a) that expresses sorbate–sorbate exclusion.

Our theory can be applied to sorbates in gas and solution alike without a need for distinguishing adsorption and dissolution/penetration. The key difference is in the interpretation of *N*_22_. While *N*_22_ for sorbent/gas is simply the sorbate–sorbate interaction, *N*_22_ for sorbent/solution is the net self-interaction, i.e., the difference between the self- (sorbate–sorbate and solvent–solvent) and mutual- (sorbate–solvent) interaction that can be considered as the generalization of the Flory χ. The relationship to sorption hysteresis will be clarified in a forthcoming paper.

## Supporting Information Available

Limiting excess number and cooperativity; derivation of the cooperative isotherm for the sorbent/solution interface; penetration of solvent and sorbate into sorbent; isotherms via differential equations; and divergent cooperativity via the differential equation approach (PDF)
