# Supplementary material for: Cooperativity in Sorption Isotherms
Source: Langmuir. 2023 Sep 22;39(39):13820–9. doi: 10.1021/acs.langmuir.3c01243 (PMC10552535; doi:10.1021/acs.langmuir.3c01243)
Supplement: Supplementary file 1 — la3c01243_si_001.pdf [file la3c01243_si_001.pdf]

Supporting information

Cooperativity in Sorption Isotherms

Seishi Shimizu[1,*] and Nobuyuki Matubayasi[2]
[1]York Structural Biology Laboratory, Department of Chemistry, University of York, Heslington, York YO10 5DD, United Kingdom.
[2]Division of Chemical Engineering, Graduate School of Engineering Science, Osaka University, Toyonaka, Osaka 560-8531, Japan

Corresponding Author: Seishi Shimizu: York Structural Biology Laboratory, Department of Chemistry, University of York, Heslington, York YO10 5DD, United Kingdom.
Email: seishi.shimizu@york.ac.uk

Table of Contents

A. Limiting excess number and cooperativity. p.S1
B. Derivation of the cooperative isotherm for the sorbent/solution interface. p.S3
C. Penetration of solvent and sorbate into sorbent. p.S4.
D. Isotherms via differential equations. p.S5.
E. Divergent cooperativity via the differential equation approach. p.S7.

A. Limiting excess number and cooperativity.

Here we prove that sorption cooperativity should strictly tend to zero at the $a_2 \to 0$ limit. In the sorbent/gas interface, the sorbate-sorbate Kirkwood-Buff integral (KBI) must converge at this limit. Let this limiting value be G_{22}^o. Then, the sorbate excess number, N_{22}, at this limit becomes 0. This can be proven using the relationship between N_{22} and G_{22} as in the following:

$$(N_{22})_{a_2\to 0} = \left(\frac{\langle n_2\rangle}{v} G_{22}\right)_{a_2\to 0} = \left(\frac{\langle n_2\rangle}{v}\right)_{a_2\to 0} G_{22}^o = 0 \quad \text{(A1)}$$

It follows that the limiting cluster number, $(N_{22}+1)_{a_2\to 0}$ is 1, which corresponds to $m = 1$. Consequently, the value for m in the cooperative characteristic relationship (eq 5) is not the limiting value at $a_2 \to 0$ but is an extrapolated value based on the finite a_2 behavior.

We can prove a similar result for the sorbent/solution interface, based on the requirement that the KBIs should not diverge at the $x_2 \to 0$ limit. To do so, let us use the following expression derived in **B. Derivation of the cooperative isotherm for the sorbent/solution interface**:

$$N_{22} + 1 = \frac{\langle n_2\rangle}{V}\left[G_{22} + G_{11} - 2G_{12} + \frac{V}{\langle n_1\rangle} + \frac{V}{\langle n_2\rangle}\right] \quad \text{(A2)}$$

which is valid both for $*$ and II. Since KBIs converge at the $a_2 \to 0$ limit and $C_2 \to 0$ at this limit, we can prove that $N_{22} + 1 \to 1$.

The limiting behavior of $N_{22} + 1$, in classical adsorption thermodynamics, has been known as the consequence of Henry's Law for sorbent/gas interface, which can be expressed at the $a_2 \to 0$ limit as

$$\langle n_2 \rangle = k_H a_2 \quad \text{(A3)}$$

where k_H is Henry's law constant. Substituting eq A3 into eq 2 leads to $N_{22} = 1$ at this limit. Note that Henry's law-based discussion is limited to sorbent/gas interfaces.

To be strictly compliant with this limiting behavior at $a_2 \to 0$, we have added, in our previous papers, the linear term $(A_1 a_2)$ to the cooperative isotherm, such that

$$\langle n_2 \rangle = N \frac{A_1 a_2 + m A_m a_2^m}{1 + A_1 a_2 + A_m a_2^m} \quad \text{(A4)}$$

Such a modification could also be introduced for the sorbent/solution isotherm. However, we shall neglect this linear term in this paper for the following reasons. Firstly, the addition of the linear term complicates the equation away from the simple, tractable form of eq 7. Secondly, the practical analysis of sorption isotherms often focuses on low a_2 behavior away from $a_2 \to 0$. This is true, particularly for the sorbent/solution interfaces for which sorption isotherm becomes increasingly difficult to measure as the sorbate concentration goes down. Note also that the amount of sorption is very small at $a_2 \to 0$ and $x_2 \to 0$, making the error introduced by omitting the linear term negligible. For these reasons, we shall focus on the low and finite a_2, instead of the limiting behavior, in this paper.

The cooperative isotherm for solid/gas and solid/vapor interfaces (eq A4) has a track record of applications to experimental isotherms. The examples include (1) water vapor adsorption isotherms on hydrophobic activated carbon fibers with pore widths of 0.5 and 0.6 nm, (2) water vapor adsorption isotherms on the pitch resin-based activated carbon materials with slit sizes of 0.5, 0.6, 1.0, and 1.1 nm.[1] Extension to multiple types of microscopic patches leads to the linear sum of eq A4 with different m and A_m.[2] This has enable to apply our theory to heterogeneous materials with multiple pore sizes, including (3) NH_3 adsorption on a hydrogen-bonded organic framework, Kuf-1a,[2] (4) water vapor adsorption on an aluminophosphate molecular sieve,[2] and (5) CO_2 adsorption on a metal-organic framework, PCN-53.[2] The

determination of sorbate cluster number and the free energy of sorption from these isotherms has led to mechanistic insights.[2]"

B. Derivation of the cooperative isotherm for the sorbent/solution interface.

Here we derive a solution-phase analogue to eq 2. The derivation can be facilitated significantly via statistical variable transformation.[3,4] As a preparation, here we show how the number variance in the $\{T, v, n_u, \mu_1, \mu_2\}$ ensemble can be converted to that in the $\{T, v, n_u, n_1, \mu_2\}$ ensemble under the invariance of the mole ratio and its fluctuation, via

$$\frac{n_2+(\delta n_2)_{\{\mu_1\}}}{n_1+(\delta n_1)_{\{\mu_1\}}} = \frac{n_2+(\delta n_2)_{\{n_1\}}}{n_1} \tag{B1a}$$

where $\{\mu_1\}$ and $\{n_1\}$ have been used for the shorthand notations for $\{T, v, n_u, \mu_1, \mu_2\}$ and $\{T, v, n_u, n_1, \mu_2\}$, respectively. Carrying out the Maclaurin expansion of eq B1a yields

$$(\delta n_2 - C_2 \delta n_1)_{\{\mu_1\}} = (\delta n_2)_{\{n_1\}} \tag{B1b}$$

where $C_2 = \langle n_2 \rangle / \langle n_1 \rangle$. This ensemble transformation (eq B1b) applies both to the interface $(*)$ and solution (II).

With the preparation above, let us carry out a differentiation of $\Gamma_2^{(1)}$ with respect to $\ln a_2$, as required for a sorbent/solution generalization of eq 2. This differentiation can be carried out straightforwardly under constant n_1, which yields

$$\left(\frac{\partial \Gamma_2^{(1)}}{\partial \ln a_2}\right)_T = \langle \delta n_2^* \delta n_2^* \rangle_{\{n_1^*\}} - \frac{n_1^*}{n_1^{II}} \langle \delta n_2^{II} \delta n_2^{II} \rangle_{\{n_1^{II}\}} \tag{B2}$$

where we have expressed the constant n_1 ensembles adopted therein emphatically by $\{n_1^*\}$ and $\{n_1^{II}\}$. Analogous to eq 1, we can introduce the excess numbers for the interface and the solution phases, N_{22}^* and N_{22}^{II}, defined in the constant n_1 ensemble, via

$$N_{22} + 1 = \frac{\langle \delta n_2 \delta n_2 \rangle_{\{n_1\}}}{\langle n_2 \rangle_{\{n_1\}}} \tag{B3}$$

and using it to rewrite eq B2 as

$$\left(\frac{\partial \Gamma_2^{(1)}}{\partial \ln a_2}\right)_T = \langle n_2^* \rangle (N_{22}^* + 1) - \frac{\langle n_1^* \rangle}{\langle n_1^{II} \rangle} \langle n_2^{II} \rangle (N_{22}^{II} + 1) \tag{B4}$$

Dividing both sides with $\Gamma_2^{(1)}$ transforms eq B4 into the following form:

$$\left(\frac{\partial \ln \Gamma_2^{(1)}}{\partial \ln a_2}\right)_T = \frac{K_e(N_{22}^*+1)-(N_{22}^{II}+1)}{K_e-1} \tag{B5}$$

where $K_e = C_2^*/C_2^{II}$ is the sorbate-solvent swapping constant.

Here we carry out the statistical variable transformation[3,4] to express $N_{22}^{*}+1$ and $N_{22}^{II}+1$ in eq B3 in terms of the fluctuations in the $\{T, v, n_u, n_1, \mu_2\}$ ensemble. This can be carried out using eq B1b as

$$N_{22}+1=\frac{\langle(\delta n_2-C_2\delta n_1)^2\rangle}{\langle n_2\rangle} \quad \text{(B6a)}$$

where the right-hand side can be expanded as

$$\frac{\langle(\delta n_2-C_2\delta n_1)^2\rangle}{\langle n_2\rangle}=\frac{\langle n_2\rangle}{v}\left[G_{22}+G_{11}-2G_{12}+\frac{v}{\langle n_1\rangle}+\frac{v}{\langle n_2\rangle}\right] \quad \text{(B6b)}$$

which represents the difference between self- and mutual interactions.

C. Penetration of solvent and sorbate into sorbent.

Let us consider a three-component system consisting of sorbent (species u), solvent (1) and sorbate (2). The system forms two phases: sorbent and solution phases. The only constraint here is that the sorbent molecules are absent in phase II. According to the Gibbs phase rule, this there-component system forming two phases has $F=3-2+2=3$ degrees of freedom.

Under constant temperature and pressure, the Gibbs-Duhem equations for the entire system $(*)$ and the reference solution phase (II), defined in the same way as in the main text,[5] are

$$\langle N_u^*\rangle d\mu_u+\langle N_1^*\rangle d\mu_1+\langle N_2^*\rangle d\mu_2=0 \quad \text{(C1)}$$

$$\langle N_u^{II}\rangle d\mu_u+\langle N_1^{II}\rangle d\mu_1+\langle N_2^{II}\rangle d\mu_2=0 \quad \text{(C2)}$$

Our condition, that the sorbent molecules are absent in the solution phase, can be expressed as

$$\langle N_u^{II}\rangle=0 \quad \text{(C3)}$$

which serves as the alternative for the Gibbs dividing surface condition. Under this condition (eq C3), subtracting eq C2 from eq C1 yields

$$\langle N_u^*\rangle d\mu_u+(\langle N_1^*\rangle-\langle N_1^{II}\rangle)d\mu_1+(\langle N_2^*\rangle-\langle N_2^{II}\rangle)d\mu_2=0 \quad \text{(C4)}$$

Under the same condition (eq C3), we obtain from eq C2

$$\left(\frac{\partial\mu_1}{\partial\mu_2}\right)_{T,P;\langle N_u^{II}\rangle=0}=-\frac{\langle N_2^{II}\rangle}{\langle N_1^{II}\rangle}=-C_2^{II} \quad \text{(C5)}$$

Combining eqs C4 and C5, we obtain

$$-\left(\frac{\partial\mu_u}{\partial\mu_2}\right)_{T,P;N_u^{II}=0}=\frac{\langle N_2^*\rangle}{\langle N_u^*\rangle}-C_2^{II}\frac{\langle N_1^*\rangle}{\langle N_u^*\rangle}=\Gamma_2^{(1)} \quad \text{(C6)}$$

The second equality in eq C6 comes from the fact that $\Gamma_2^{(1)}=\langle n_2^*\rangle-C_2^{II}\langle n_1^*\rangle$ is commonly reported per the unit quantity of sorbent.

To summarize, we have derived eq C6 for the sorbent-solution phase equilibrium under the only condition that the sorbent molecules do not dissolve into the solution phase. This means that eq C6 (eq 8a) is valid even when solvent and sorbate molecules dissolve into or penetrate the sorbent. Consequently, starting from $\Gamma_2^{(1)}$ introduced in eq C6, we can follow the discussion as presented in **B. Derivation of the cooperative isotherm for the sorbent/solution interface** to derive our fundamental equations, eq 9a for sorbent/solution and eq 2 for sorbent/gas sorption where the latter can be derived as the special case of eq 9a. Note that the connection to the discussions in **B. Derivation of the cooperative isotherm for the sorbent/solution interface**, carried out in a constant n_u ensemble, can be made seamlessly by the fact that $\Gamma_2^{(1)}$ is an intensive quantity independent of the choice of the ensemble.

We have previously discussed the similarity and differences between (a) the preferential solvation (involving a solute in dilution) and (b) the Gibbs adsorption isotherm (of an interface in a system of a two-component mixture).[6] The difference between the two within this setup was rationalized by the Gibbs phase rule. The solute, in the case of the preferential solvation (a), does not contribute to the degrees of freedom because it is at infinite dilution. However, the presence of the interface in (b) reduces the degrees of freedom in the context of the surface excess. In contrast to the above comparison, the discussion in this section shows the parallel between (c) the preferential solvation of a solute at infinite dilution in an n-component solution and (d) sorption from an n-component solution mixture species onto a sorbent that is absent in the solution. In comparison to (c), (d) has one more degree of freedom in the Gibbs phase rule by the introduction of the sorbent, which is yet is reduced by one due to the presence of an interface. The absence of the sorbent in the solution in (d) corresponds to the absence of the infinitely dilute solute in the bulk in (c).

D. Isotherms via differential equations.

The ABC Isotherm. The ABC isotherm is a model-free isotherm that encompasses the Langmuir, BET, and GAB models as its special cases.[7–9] Its derivation follows the same logical steps as the cooperative isotherm summarized in the main text.

- the fundamental equation, which has been rewritten using the sorbate-sorbate Kirkwood-Buff integral, $\frac{G_{22}}{v} = \frac{N_{22}}{\langle n_2 \rangle}$, as

$$\left(\frac{\partial}{\partial a_2}\frac{a_2}{\langle n_2 \rangle}\right)_T = -\frac{G_{22}}{v} \tag{D1a}$$

- the characteristic relationship for sorbate fluctuation, stating how G_{22} depends on sorbate activity, via

$$\frac{G_{22}}{v} = B + Ca_2 \tag{D1b}$$

Integrating eq D1a in combination with eq D1b yields

$$\langle n_2 \rangle = \frac{a_2}{A - Ba_2 - \frac{C}{2}a_2^2} \tag{D1c}$$

The parameters A, B, and C have a clear statistical thermodynamic interpretation in terms of the mono-, di-, and tri-sorbate Kirkwood-Buff integrals, as has been shown in our recent papers.[7–9] This isotherm has also been generalized to sorbent/solution interfaces.

The Cubic Isotherm. Here we start with a different expression for the fundamental equation,

$$\left(\frac{\partial}{\partial a_2}\frac{\langle n_2 \rangle}{a_2}\right)_T = \frac{\langle n_2 \rangle^2}{a_2^2}\frac{G_{22}}{v} \tag{D2a}$$

which can be derived straightforwardly from eq D1a, in combination with a different characteristic relationship,

$$\frac{\langle n_2 \rangle^2}{a_2^2}\frac{G_{22}}{v} = B' + C'a_2 \tag{D2b}$$

Integrating eq D2a with eq D2b yields the following isotherm:

$$\langle n_2 \rangle = A'a_2 + B'a_2^2 + \frac{C'}{2}a_2^3 \tag{D2c}$$

where A' was introduced upon integration. This is the polynomial isotherm founded upon sorbate number correlation and the meaning of its parameters A', B', and C', will be discussed below. The parameters A', B', and C' can be interpreted by comparing eq D2c with the Maclaurin expansion of the ABC isotherm (eq D1c), which yields

$$A' = \frac{1}{A},\ \ B' = \frac{B}{A^2},\ \ C' = \frac{C}{A^2} + \frac{2B^2}{A^3} \tag{D2d}$$

through which the parameters can be expressed using KBIs.

The Virial Isotherm. Here we adopt eq D2a again for the fundamental equation yet with a characteristic relationship different from eq D2a, as

$$\frac{N_{22}}{a_2} = B'' + C''a_2 \tag{D3a}$$

Integrating eq D2a along with eq D3a, we obtain

$$\langle n_2 \rangle = A''a_2 e^{\left(B''a_2 + \frac{C''}{2}a_2^2\right)} \tag{D3b}$$

where the constant A'' was introduced upon integration. This isotherm has been known as the virial isotherm.[10,11] The parameters of the exponential isotherm can be related to those of the ABC isotherm

$$\langle n_2\rangle = A''a_2 + A''B''a_2^2 + \frac{1}{2}A''\left(B''^2 + C''\right)a_2^3 + \cdots \tag{D3c}$$

$$\langle n_2\rangle = \frac{a_2}{A-Ba_2-\frac{C}{2}a_2^2} = \frac{1}{A}a_2 + \frac{B}{A^2}a_2^2 + \frac{1}{A}\left(\frac{B^2}{A^2} + \frac{C}{2A}\right)a_2^3 + \cdots \tag{D3d}$$

The comparison of eqs D3c and D3d yields

$$A'' = \frac{1}{A} \tag{D3e}$$

$$B'' = \frac{1}{A''}\frac{B}{A^2} = \frac{B}{A} \tag{D3f}$$

$$C'' = \frac{C}{A} + \left(\frac{B}{A}\right)^2 \tag{D3g}$$

A'', B'' and C'' have a clear link to A, B, and C , hence to the sorbate-surface, sorbate pair, and triplet interactions.[9]

E. Divergent cooperativity via the differential equation approach.

Here we show that the AB isotherm for divergent cooperativity can be derived from the fluctuation equation (eq 2) in combination with the characteristic relationship (eq 14c) that signifies a linearly increasing sorbate cluster number. Combining eqs 2 and 14c under constant T yields the following differential equation:

$$\frac{d\langle n_2\rangle}{\langle n_2\rangle(1+B\langle n_2\rangle)} = d\ln a_2 \tag{E1}$$

Partial fraction decomposition yields

$$\frac{1}{\langle n_2\rangle(1+B\langle n_2\rangle)} = \frac{1}{\langle n_2\rangle} - \frac{1}{\langle n_2\rangle+\frac{1}{B}} \tag{E2}$$

Combining eqs E1 and E2 and integrating them yields

$$\ln\frac{\langle n_2\rangle}{\langle n_2\rangle+\frac{1}{B}} = \ln ka_2 \tag{E3}$$

where k is an integration constant. Solving eq E3 for $\langle n_2\rangle$ yields

$$\langle n_2\rangle = \frac{a_2}{\frac{B}{k}-Ba_2} \tag{E4}$$

Putting $\frac{B}{k} = A$ leads to the AB isotherm.

References

(1) Shimizu, S.; Matubayasi, N. Cooperative Sorption on Porous Materials. *Langmuir* **2021**, *37* (34), 10279–10290. https://doi.org/10.1021/acs.langmuir.1c01236.

(2) Dalby, O.; Abbott, S.; Matubayasi, N.; Shimizu, S. Cooperative Sorption on Heterogeneous Surfaces. *Langmuir* **2022**, *38* (43), 13084–13092. https://doi.org/10.1021/acs.langmuir.2c01750.

(3) Shimizu, S.; Matubayasi, N. Intensive Nature of Fluctuations: Reconceptualizing Kirkwood-Buff Theory via Elementary Algebra. *J. Mol. Liq.* **2020**, *318*, 114225. https://doi.org/10.1016/j.molliq.2020.114225.

(4) Shimizu, S.; Matubayasi, N. Ensemble Transformation in the Fluctuation Theory. *Physica A* **2022**, *585*, 126430. https://doi.org/10.1016/J.PHYSA.2021.126430.

(5) Shimizu, S.; Matubayasi, N. Fluctuation Adsorption Theory: Quantifying Adsorbate-Adsorbate Interaction and Interfacial Phase Transition from an Isotherm. *Phys. Chem. Chem. Phys.* **2020**, *22*, 28304–28316. https://doi.org/10.1039/D0CP05122E.

(6) Shimizu, S.; Matubayasi, N. Preferential Solvation: Dividing Surface vs Excess Numbers. *J. Phys. Chem. B* **2014**, *118*, 3922–3930. https://doi.org/10.1021/jp410567c.

(7) Shimizu, S.; Matubayasi, N. Sorption: A Statistical Thermodynamic Fluctuation Theory. *Langmuir* **2021**, *37*, 7380–7391. https://doi.org/10.1021/acs.langmuir.1c00742.

(8) Shimizu, S.; Matubayasi, N. Surface Area Estimation: Replacing the BET Model with the Statistical Thermodynamic Fluctuation Theory. *Langmuir* **2022**, *38*, 7989–8002. https://doi.org/10.1021/acs.langmuir.2c00753.

(9) Shimizu, S.; Matubayasi, N. Understanding Sorption Mechanisms Directly from Isotherms. *Langmuir* **2023**, *39* (17), 6113–6125. https://doi.org/10.1021/acs.langmuir.3c00256.

(10) Barrer, R. M.; Davies, J. A. Sorption in Decationated Zeolites. I. Gases in Hydrogen-Chabazite. *Proc. R. Soc. London. A. Math. Phys. Sci.* **1970**, *320* (1542), 289–308. https://doi.org/10.1098/rspa.1970.0210.

(11) Ruthven, D. M. Sorption Kinetics for Diffusion-Controlled Systems with a Strongly Concentration-Dependent Diffusivity. *Chem. Eng. Sci.* **2004**, *59* (21), 4531–4545. https://doi.org/10.1016/j.ces.2004.06.028.
